# Aerosolized antifungal therapy in pulmonary fungal disease: pharmacology, drug delivery and emerging clinical applications

**DOI:** 10.3389/fmed.2026.1937682

**Published:** 2026-09-03

**Authors:** Massimo Caracciolo, Sarah Antonella Caracciolo, Maria Francesca Stagno, Simona Pellicano, Antonino Ripepi, Nadia Pellicano, Clara Scopelliti, Angela Elvira Serranò, Antonino Loddo, Stefano La Scala

**Affiliations:** 1 Anesthesia and Post-Operative Care Unit, Villa Sant'Anna Hospital, Reggio Calabria, Italy; 2 Department of Anesthesia and Intensive Care, Santa Maria Nuova Hospital, Reggio Emilia, Italy; 3 UOSD Post-Operative Intensive Care Unit, Great Metropolitan Hospital “Bianchi Melacrino Morelli”, Reggio Calabria, Italy

**Keywords:** invasive pulmonary aspergillosis, pulmonary fungal disease, aerosolized antifungal therapy, chronic pulmonary aspergillosis, inhaled antifungal therapy, lung transplantation, nebulization, vibrating mesh nebulizer, pulmonary drug delivery

## Abstract

Pulmonary fungal diseases remain associated with substantial morbidity and mortality, particularly in immunocompromised individuals, transplant recipients, and critically ill patients. Although systemic antifungal therapy remains the cornerstone of treatment, its effectiveness may be limited by suboptimal penetration into selected pulmonary compartments, while systemic toxicity, drug–drug interactions, and prolonged treatment requirements may restrict therapeutic options. Aerosolized antifungal therapy provides a pharmacologically attractive approach to increase local pulmonary drug exposure while limiting systemic exposure. Among currently available formulations, liposomal amphotericin B has the most extensive clinical experience for inhaled administration, supported by favorable pulmonary retention, respiratory tolerability, and compatibility with contemporary nebulization technologies. Advances in vibrating mesh nebulizers and aerosol delivery during invasive mechanical ventilation and high-flow respiratory support have further improved the technical feasibility of targeted pulmonary drug administration. Current clinical evidence is strongest for prophylaxis of invasive pulmonary aspergillosis in selected high-risk populations, particularly patients with prolonged neutropenia. Aerosolized amphotericin B formulations are also incorporated into prophylactic strategies after lung transplantation, although protocols remain heterogeneous and evidence is predominantly observational. By contrast, therapeutic applications beyond prophylaxis remain investigational. The potential role of aerosolized therapy differs substantially among pulmonary fungal diseases according to pathogen biology, anatomical distribution, host characteristics, and accessibility of the affected pulmonary compartment. Chronic pulmonary aspergillosis may provide a rationale for localized drug delivery, but cavitary disease and heterogeneous ventilation may limit aerosol deposition. For pulmonary mucormycosis, cryptococcosis, *Pneumocystis jirovecii* pneumonia, and endemic mycoses, current evidence is insufficient to support routine inhaled antifungal therapy. This Mini Review summarizes current evidence on aerosolized antifungal formulations, pulmonary pharmacology, nebulization technologies, and disease-specific clinical applications, while highlighting current limitations and priorities for future translational and clinical research.

## 1 Introduction

Pulmonary fungal diseases represent a heterogeneous group of infections associated with substantial morbidity and mortality, particularly among immunocompromised individuals, transplant recipients, and critically ill patients (38). Their clinical spectrum ranges from acute invasive pulmonary aspergillosis (IPA) to chronic pulmonary aspergillosis (CPA), airway-predominant fungal disease, and pulmonary involvement caused by other opportunistic or endemic fungi (39, 40). These conditions differ substantially in pathogenesis, anatomical distribution, host susceptibility, and therapeutic requirements, making a disease-specific approach essential when considering pulmonary antifungal strategies.

**Table 1:** Principal antifungal agents and formulations evaluated or developed for pulmonary administration.

Antifungal agent/formulation	Formulation characteristics	Inhaled regulatory/developmental status	Main pulmonary evidence	Potential advantages	Main limitations
Amphotericin B deoxycholate (AmB-d)	Conventional amphotericin B formulation; can be nebulized but was not specifically developed for pulmonary administration	Off-label when inhaled; no approved inhaled formulation	Historical clinical experience, particularly for prophylaxis; heterogeneous dosing and delivery protocols (2–4)	Broad antifungal spectrum; extensive historical experience	Airway irritation, cough and bronchospasm; less favorable pulmonary tolerability than lipid formulations; heterogeneous aerosol characteristics
Liposomal amphotericin B (L-AmB)	Liposomal amphotericin B formulation with prolonged pulmonary retention and reduced epithelial toxicity compared with conventional amphotericin B	Off-label when inhaled; no approved inhaled formulation	Most extensive clinical experience among inhaled antifungal formulations; randomized evidence for IPA prophylaxis during prolonged neutropenia and observational experience after lung transplantation (1–4)	High local pulmonary exposure; prolonged lung retention; limited systemic exposure; generally favorable respiratory tolerability; broad antifungal spectrum	Heterogeneous dosing schedules and nebulization protocols; variable deposition according to disease and device; therapeutic efficacy in established fungal disease remains insufficiently defined
Voriconazole	Systemic triazole formulation experimentally adapted for nebulization; no dedicated commercially approved inhaled preparation	Investigational/off-label when aerosolized; no approved inhaled formulation	Limited experimental and early clinical experience	Mold-active triazole; theoretical potential for targeted airway delivery	Formulation and aerosolization challenges; limited pulmonary PK and clinical data; no established inhaled dosing regimen
Itraconazole—PUR1900	Dry-powder formulation of itraconazole specifically developed for targeted pulmonary delivery	Investigational dedicated inhaled formulation; not approved for routine clinical use	Randomized, double-blind, placebo-controlled phase 2 trial in adults with asthma and ABPA; proof-of-concept clinical evidence (43)	Targeted airway delivery; reduced systemic exposure; potential to limit systemic azole-related toxicity and drug–drug interactions; randomized phase 2 clinical evidence	Small exploratory phase 2 trial; no prespecified primary efficacy endpoint; clinical efficacy not yet established; confirmatory trials required
Posaconazole—inhaled formulations	Nanocrystal and dry-powder formulations developed to increase local pulmonary exposure	Preclinical/investigational; no approved inhaled formulation	Favorable pulmonary deposition and pharmacokinetic characteristics demonstrated predominantly in preclinical models (41)	Potential for high local concentrations and prolonged pulmonary exposure with reduced systemic exposure	Clinical efficacy and safety not established; formulation–device performance and human dosing require validation
Opelconazole (PC945)	Triazole specifically designed for inhaled pulmonary administration with prolonged local retention and limited systemic absorption	Investigational dedicated inhaled antifungal; clinical development ongoing	Preclinical and early clinical-development evidence, including limited clinical experience in respiratory *Aspergillus* disease (31–33)	Lung-targeted exposure; prolonged pulmonary residence; limited systemic exposure and potentially fewer systemic drug interactions	Controlled efficacy data remain limited; optimal indications, dosing, long-term safety, and comparative effectiveness remain undefined
Echinocandins	Large lipopeptide molecules; no clinically established formulation specifically optimized for inhalation	Preclinical/experimental; no approved inhaled formulation	Limited aerosol and pulmonary PK studies; no established clinical role (7, 11)	Theoretical potential for local activity against susceptible fungi and biofilm-associated organisms	Limited aerosol-development data; uncertain epithelial penetration and regional deposition; absence of dedicated inhaled formulations and clinical efficacy data

Principal antifungal agents and formulations evaluated or specifically developed for pulmonary administration. Liposomal amphotericin B has the most extensive clinical experience with aerosol delivery, although inhaled administration remains off-label and no dedicated inhaled formulation is currently approved for routine clinical use. Dedicated pulmonary triazole formulations are at different stages of development: PUR1900 has recently provided randomized phase 2 proof-of-concept evidence in allergic bronchopulmonary aspergillosis, whereas opelconazole remains under clinical development and inhaled posaconazole formulations are supported predominantly by preclinical data. Regulatory/developmental status refers specifically to pulmonary administration and should not be interpreted as the regulatory status of the corresponding systemic antifungal agent. Technical aerosolizability, favorable pulmonary pharmacokinetics, or successful local drug deposition should not be interpreted as evidence of clinical efficacy. ABPA, allergic bronchopulmonary aspergillosis; AmB-d, amphotericin B deoxycholate; IPA, invasive pulmonary aspergillosis; L-AmB, liposomal amphotericin B; PK, pharmacokinetics.

Systemic antifungal therapy remains the cornerstone of treatment for invasive and chronic pulmonary fungal diseases. However, achieving adequate antifungal exposure at the site of infection may be challenging because pulmonary drug distribution is influenced by tissue vascularization, inflammation, necrosis, airway obstruction, cavitation, and the physicochemical properties of individual antifungal agents (11–14). Systemic toxicity, drug–drug interactions, and prolonged treatment requirements may further limit therapeutic optimization in selected patients.

These limitations have renewed interest in aerosolized antifungal therapy as a strategy to deliver high local drug concentrations directly to airway-accessible pulmonary compartments while reducing systemic exposure (2–4). Among currently available formulations, aerosolized liposomal amphotericin B (L-AmB) has generated the most extensive clinical experience. Randomized and observational evidence supports its use primarily for prophylaxis of IPA in selected high-risk populations, particularly patients with prolonged neutropenia (1–4). Aerosolized amphotericin B formulations are also incorporated into prophylactic strategies after lung transplantation, although protocols remain heterogeneous and no single approach is currently supported as universally optimal (42).

By contrast, the therapeutic role of aerosolized antifungal agents beyond prophylaxis remains considerably less established. Importantly, the biological and pharmacological rationale for inhaled therapy cannot be generalized across pulmonary fungal diseases. Potential benefit depends not only on antifungal activity but also on the anatomical accessibility of the infected compartment to inhaled particles. Airway-predominant or superficially accessible disease may theoretically be more amenable to aerosol delivery, whereas poorly ventilated lesions, thick-walled cavities, vascular or angioinvasive infection, pleural disease, and disseminated fungal infection may substantially limit its effectiveness or require systemic therapy as the primary therapeutic approach.

This distinction is particularly relevant across the spectrum of pulmonary aspergillosis. IPA, CPA, and airway aspergillosis represent biologically and anatomically distinct conditions (39, 40), and evidence supporting aerosolized prophylaxis against IPA cannot be directly extrapolated to treatment of established invasive or chronic disease. Similar caution applies to pulmonary mucormycosis, cryptococcosis, *Pneumocystis jirovecii* pneumonia, and endemic pulmonary mycoses, for which disease distribution, pathogen biology, and available clinical evidence differ substantially.

Respiratory isolation of *Candida* species represents a separate issue. In non-neutropenic critically ill patients, isolation of *Candida* from respiratory specimens is generally interpreted as colonization rather than invasive pulmonary candidiasis and does not, by itself, justify antifungal treatment (5, 7, 9, 10). Although persistent fungal airway carriage and host–fungus interactions remain areas of biological interest, their clinical significance and potential relevance to targeted pulmonary antifungal therapy remain unproven and should be considered research questions rather than therapeutic indications.

The present Mini Review summarizes the pharmacological basis of aerosolized antifungal therapy, examines currently available and investigational inhaled antifungal formulations, reviews advances in aerosol delivery during spontaneous breathing and respiratory support, and critically evaluates clinical applications using a disease-oriented framework. Particular emphasis is placed on distinguishing evidence-supported prophylactic applications from investigational therapeutic strategies and on defining the anatomical, pharmacological, and clinical limitations that may determine whether aerosolized antifungal therapy is appropriate for specific pulmonary fungal diseases.

## 2 Literature search strategy

A focused literature search was conducted in PubMed and Scopus for publications available from January 1990 through July 2026. Search terms included combinations of “aerosolized antifungal therapy,” “inhaled antifungal therapy,” “inhaled amphotericin B,” “liposomal amphotericin B,” “pulmonary fungal disease,” “invasive pulmonary aspergillosis,” “chronic pulmonary aspergillosis,” “allergic bronchopulmonary aspergillosis,” “ABPA,” “inhaled itraconazole,” “PUR1900,” “lung transplantation,” “mucormycosis,” “cryptococcosis,” “Pneumocystis jirovecii,” “endemic mycoses,” “nebulization,” “vibrating mesh nebulizer,” “mechanical ventilation,” “high-flow nasal oxygen,” “pulmonary drug delivery,” “pulmonary pharmacokinetics,” and “fungal biofilm.”

English-language original studies, randomized and non-randomized clinical trials, observational studies, pharmacokinetic and aerosol-delivery studies, systematic and narrative reviews, international guidelines, and clinically relevant experimental studies were considered. Particular attention was given to studies evaluating aerosolized antifungal formulations, pulmonary drug deposition and pharmacokinetics, prophylactic and therapeutic applications, and disease-specific factors potentially influencing the effectiveness of inhaled drug delivery.

Reference lists of selected publications were also screened to identify additional relevant studies. Priority was given to clinical evidence, international guidelines, and studies directly addressing aerosolized antifungal therapy; experimental and mechanistic studies were included when necessary to discuss pharmacological rationale, formulation characteristics, aerosol delivery, or emerging research questions.

Because this article was designed as a structured narrative Mini Review, study selection was not performed according to systematic-review methodology. No formal PRISMA-based screening process, predefined risk-of-bias assessment, or quantitative evidence synthesis was undertaken.

## 3 Pharmacological basis of aerosolized antifungal therapy

The rationale for aerosolized antifungal therapy derives from the pharmacokinetic and anatomical challenges associated with pulmonary fungal disease. Systemically administered antifungal agents must reach effective concentrations within pulmonary sites that may differ substantially in vascularization, inflammation, ventilation, tissue architecture, and accessibility. Consequently, adequate plasma concentrations do not necessarily ensure optimal drug exposure at every site of pulmonary fungal infection (6, 11–14).

Pulmonary drug distribution is particularly heterogeneous in structurally abnormal or severely diseased lungs. Airway secretions, mucus plugs, distal bronchioles, alveolar spaces, necrotic lesions, cavitary disease, and biofilm-containing airway surfaces may differ considerably in their accessibility to systemic antifungal agents. In critically ill patients, pulmonary edema, microvascular dysfunction, airway obstruction, heterogeneous ventilation–perfusion relationships, and invasive respiratory support may further modify local drug distribution.

The pharmacokinetic characteristics of systemic antifungal agents illustrate these challenges. Amphotericin B formulations achieve substantial concentrations within pulmonary tissue but may exhibit variable distribution across specific anatomical compartments (11–13). Echinocandins similarly provide reliable systemic exposure but show heterogeneous penetration into respiratory secretions and selected pulmonary sites (11, 12). These observations provide a pharmacological rationale for investigating complementary approaches capable of increasing local pulmonary antifungal exposure.

Aerosol administration allows antifungal agents to be deposited directly within the respiratory tract, potentially generating local concentrations greater than those achieved through systemic administration while limiting systemic exposure (2–4). This approach is particularly relevant for airway-accessible and adequately ventilated pulmonary regions, where inhaled particles can reach the site of fungal deposition. However, pulmonary deposition is inherently heterogeneous and depends on particle characteristics, airway geometry, regional ventilation, respiratory pattern, underlying lung disease, and the aerosol-delivery system.

Importantly, the pharmacological advantages of inhalation should not be extrapolated to pulmonary compartments that are poorly accessible from the airways. Thick-walled or poorly ventilated cavities, extensive mucus obstruction, consolidated or necrotic lung regions, pleural collections, and non-communicating postoperative cavities may receive limited or negligible aerosol deposition. Pleural collections and non-communicating cavities should therefore not be considered direct targets of inhaled antifungal therapy unless anatomical communication with the respiratory tract is present. Likewise, aerosolized therapy cannot substitute for systemic treatment when fungal disease is angioinvasive, extrapulmonary, or disseminated.

**Figure 1:**
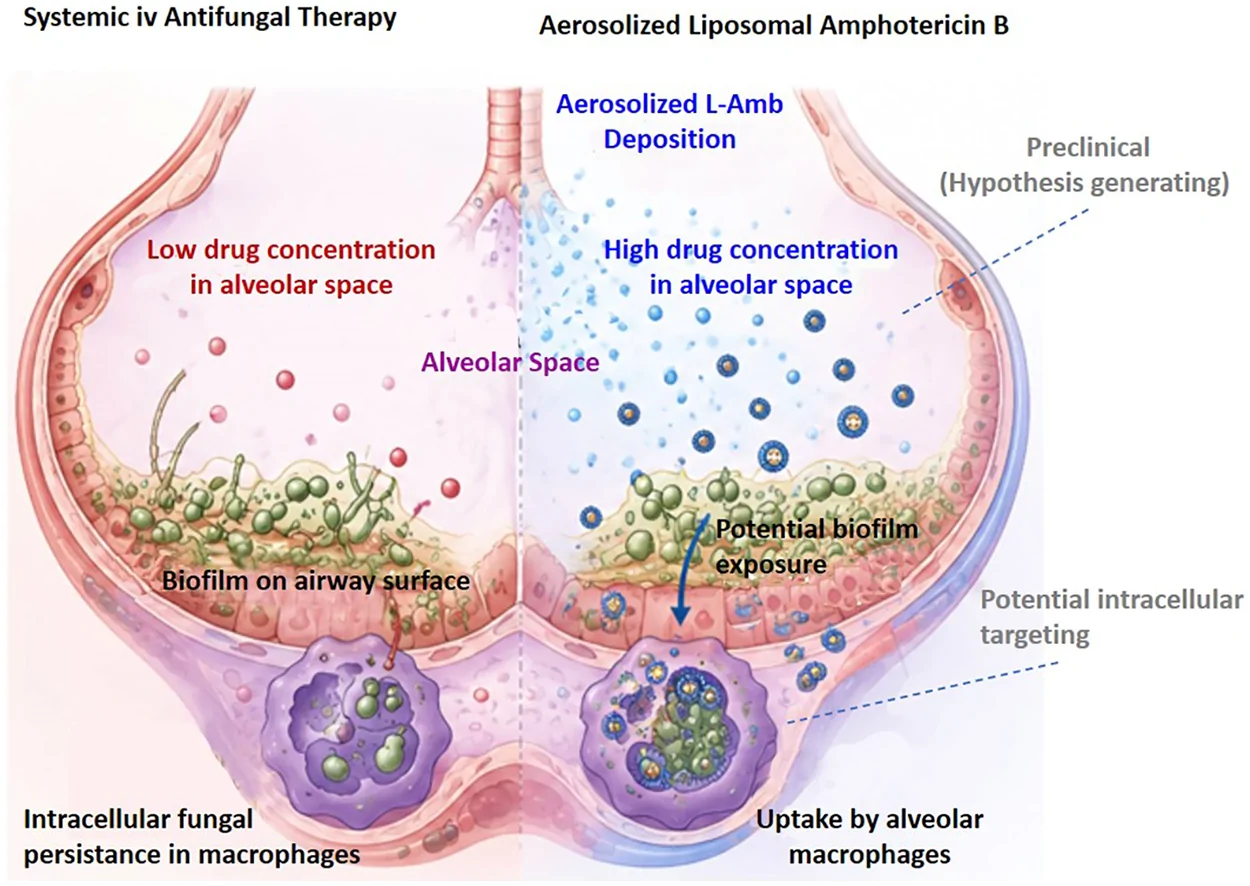
Conceptual illustration of the pharmacological rationale for aerosolized liposomal amphotericin B. Aerosol administration is intended to increase local antifungal exposure within airway-accessible pulmonary compartments while minimizing systemic exposure. Experimental studies have demonstrated uptake of liposomal amphotericin B by alveolar macrophages, suggesting the potential for intracellular drug delivery. Likewise, enhanced drug exposure within airway secretions and biofilm-containing compartments represents a biologically plausible mechanism, although its clinical relevance has not been established. The mechanisms indicated by dashed arrows and labeled as **Preclinical (Hypothesis-generating)** are supported predominantly by experimental evidence and require further clinical validation. This schematic illustration is intended to represent conceptual pharmacological principles rather than quantitative pharmacokinetic differences between systemic and inhaled administration. Current clinical evidence supports aerosolized liposomal amphotericin B primarily for prophylaxis of invasive pulmonary aspergillosis in selected high-risk patients, whereas its therapeutic role in other pulmonary fungal diseases remains investigational.

Inhaled administration may also influence local antifungal pharmacodynamics. Amphotericin B exhibits concentration-dependent fungicidal activity through binding to ergosterol within the fungal cell membrane. High local concentrations may therefore theoretically increase antifungal exposure at accessible respiratory surfaces. Experimental studies have additionally explored drug exposure within fungal biofilms and uptake of liposomal amphotericin B by alveolar macrophages. These mechanisms are biologically plausible and potentially relevant to pulmonary drug targeting, but their clinical significance has not been established and they should be regarded as preclinical or hypothesis-generating observations rather than demonstrated therapeutic advantages (Figure 1).

The pharmaceutical formulation is a critical determinant of successful aerosol administration. Amphotericin B deoxycholate retains broad antifungal activity but inhaled administration may cause cough, bronchospasm, and airway irritation, limiting tolerability in some patients (2–4). Lipid-based formulations modify the pulmonary pharmacological profile of amphotericin B by reducing direct exposure to free drug and potentially improving respiratory tolerability. Among these formulations, liposomal amphotericin B has accumulated the greatest clinical experience for aerosol administration and combines prolonged pulmonary retention with a generally favorable tolerability profile (1–4).

Preservation of formulation integrity during nebulization is particularly important for liposomal preparations. Aerosol-generation technologies may influence particle-size distribution, residual drug volume, treatment duration, and preservation of liposomal structure. Vibrating mesh nebulizers offer several technical advantages, including efficient aerosol generation and limited heating of the formulation, but these pharmacotechnical characteristics should not be interpreted as evidence of superior clinical outcomes. Formulation–device compatibility and reproducibility of the delivered dose remain important considerations for both clinical practice and future trials.

Overall, the effectiveness of aerosolized antifungal therapy depends on the interaction of three principal determinants: the pharmaceutical formulation, the efficiency of aerosol generation and pulmonary deposition, and the anatomical accessibility of the fungal disease. Liposomal amphotericin B currently has the most extensive clinical experience among aerosolized antifungal formulations, particularly for prophylactic applications. However, the potential value of inhaled therapy in established pulmonary fungal disease must be evaluated according to the specific pathogen, disease phenotype, anatomical distribution, and accessibility of the affected pulmonary compartment rather than inferred solely from the ability to achieve high local respiratory drug concentrations.

## 4 Antifungal formulations for aerosol administration

The clinical potential of aerosolized antifungal therapy depends not only on the intrinsic antifungal activity of the administered agent but also on its pharmaceutical formulation, physicochemical properties, stability during nebulization, pulmonary retention, local tolerability, and compatibility with aerosol-generation devices. Importantly, most antifungal agents currently used by inhalation were originally developed for systemic administration, and their aerosolized use is generally off-label. Dedicated inhaled antifungal formulations remain limited and are predominantly investigational. The principal antifungal agents and formulations evaluated or developed for pulmonary administration are summarized in Table 1.

### 4.1 Amphotericin B deoxycholate

Amphotericin B deoxycholate (d-AmB) was among the earliest antifungal formulations administered by inhalation. Its broad antifungal spectrum and concentration-dependent fungicidal activity provide a pharmacological rationale for local pulmonary delivery. However, aerosolized d-AmB may cause cough, unpleasant taste, bronchospasm, and airway irritation, limiting tolerability in some patients (2–4).

In addition, because d-AmB was developed for intravenous rather than inhaled administration, aerosol characteristics and delivered pulmonary dose may vary according to nebulizer type, formulation concentration, and respiratory conditions. Although d-AmB has historically been used in prophylactic protocols, particularly in transplant populations, its inhaled administration remains off-label and has increasingly been complemented or replaced in some settings by lipid-based amphotericin B formulations with improved respiratory tolerability.

### 4.2 Liposomal amphotericin B

Liposomal amphotericin B (L-AmB) has accumulated the most extensive clinical experience among lipid-based formulations used for aerosol administration. Incorporation of amphotericin B within liposomal vesicles modifies its interaction with pulmonary tissues and reduces direct exposure to free amphotericin B, contributing to improved local tolerability compared with conventional deoxycholate formulations (1–4).

Experimental and pharmacokinetic studies suggest prolonged pulmonary retention after inhaled administration, while systemic exposure generally remains limited. These properties make L-AmB particularly attractive for prophylactic strategies requiring repeated pulmonary administration.

Preservation of liposomal integrity during aerosol generation remains an important pharmacotechnical consideration. Nebulization may potentially alter vesicle structure, particle characteristics, and drug-release behavior, making formulation–device compatibility relevant to reproducibility of pulmonary delivery. Vibrating mesh nebulizers have technical characteristics that may favor administration of liposomal formulations, although evidence of pharmacotechnical compatibility should not be interpreted as evidence of superior clinical efficacy.

Clinical evidence for aerosolized L-AmB is strongest in the prevention of invasive pulmonary aspergillosis, particularly in selected patients with prolonged neutropenia (1–4). It is also used in center-specific prophylactic strategies after lung transplantation. By contrast, evidence supporting aerosolized L-AmB as treatment for established pulmonary fungal disease remains substantially weaker and should not be extrapolated from prophylactic studies.

### 4.3 Amphotericin B lipid Complex

Amphotericin B lipid complex (ABLC) represents another lipid-associated formulation that has been administered by inhalation, particularly in selected transplant settings. Its lipid-based structure may reduce local toxicity compared with conventional amphotericin B, but clinical experience with aerosolized ABLC is less extensive and standardized than that available for L-AmB.

Differences in lipid composition, particle behavior, nebulization characteristics, and pulmonary retention mean that evidence obtained with one amphotericin B formulation should not automatically be extrapolated to another. Consequently, d-AmB, L-AmB, and ABLC should be considered pharmacologically distinct aerosol formulations rather than interchangeable preparations.

### 4.4 Inhaled triazoles

Development of triazoles specifically intended or adapted for pulmonary administration represents an important area of antifungal research. Early investigations explored aerosolized voriconazole, but the absence of an approved inhaled formulation and limited clinical evidence have restricted its application.

More recently, inhaled posaconazole formulations, including nanocrystal-based dry-powder systems, have demonstrated encouraging pulmonary pharmacokinetic characteristics in preclinical studies (41). These findings support continued pharmaceutical development but do not currently establish a clinical indication for inhaled posaconazole.

Importantly, recent randomized clinical evidence has emerged for PUR1900, a dry-powder formulation of itraconazole specifically developed for inhaled pulmonary delivery. In the multicenter, randomized, double-blind, placebo-controlled phase 2 PUR1900-ABPA trial, adults with asthma and allergic bronchopulmonary aspergillosis (ABPA) received inhaled PUR1900 or placebo. The 40-mg dose group showed encouraging improvements in lung function and asthma control, together with a favorable safety profile (43). However, the exploratory design, relatively small sample size, and absence of a prespecified primary efficacy endpoint limit definitive conclusions. These findings therefore provide proof-of-concept randomized evidence supporting further clinical development rather than establishing inhaled itraconazole as standard therapy for ABPA.

Another advanced inhaled triazole is opelconazole (PC945), a compound specifically designed for pulmonary administration. Preclinical and early clinical investigations have demonstrated prolonged pulmonary exposure and limited systemic absorption, and preliminary clinical experience has supported further investigation in respiratory Aspergillus disease (31–33). However, larger controlled clinical studies are required before its therapeutic role can be established.

Accordingly, inhaled triazoles represent an emerging class of pulmonary antifungal therapies. Recent randomized evidence with PUR1900 strengthens the clinical rationale for targeted delivery in selected airway-predominant Aspergillus-related disease, but these agents should still be considered investigational and should not be regarded as established alternatives to current guideline-based systemic or anti-inflammatory treatment strategies.

### 4.5 Echinocandins

Echinocandins are established systemic agents for invasive candidiasis and have important activity against *Candida* and *Aspergillus* species, but they currently have no established clinical role as aerosolized antifungal therapy (7, 11).

Their physicochemical characteristics, absence of dedicated approved inhaled formulations, and limited pulmonary aerosol pharmacokinetic data have restricted clinical development. Experimental approaches to pulmonary delivery may eventually provide new formulation strategies, but current evidence remains insufficient to support clinical recommendations for inhaled echinocandins.

### 4.6 Emerging pulmonary antifungal formulations

Advances in pulmonary drug-delivery technology have stimulated development of dry-powder formulations, lipid carriers, nanoparticles, and other delivery systems intended to improve local pulmonary exposure while limiting systemic toxicity. These strategies may offer theoretical advantages in terms of formulation stability, pulmonary retention, and targeted delivery.

However, most remain at the preclinical or early developmental stage. Translation into clinical practice will require demonstration not only of favorable pulmonary pharmacokinetics but also of reproducible delivered dose, formulation–device compatibility, respiratory safety, and clinically meaningful efficacy.

The development of agents specifically engineered for inhalation, rather than adaptation of intravenous formulations for nebulization, may ultimately provide a more standardized approach to pulmonary antifungal therapy.

Overall, L-AmB currently has the most extensive clinical experience among aerosolized antifungal formulations, whereas dedicated inhaled triazoles and other novel pulmonary formulations remain investigational. Importantly, pharmaceutical suitability for aerosol administration does not itself establish clinical efficacy. The therapeutic value of each formulation must ultimately be evaluated according to the specific fungal disease, anatomical distribution, pulmonary accessibility, quality of clinical evidence, and intended use as prophylactic, adjunctive, or investigational therapy.

## 5 Aerosol delivery technologies

The clinical performance of inhaled antifungal therapy depends not only on the pharmaceutical formulation but also on the efficiency and reproducibility of aerosol generation and pulmonary deposition. Nebulizer technology, particle-size distribution, respiratory pattern, airway anatomy, humidification, underlying lung disease, and the mode of respiratory support may substantially influence the fraction of the nominal dose reaching the lower respiratory tract (24–30, 34–37).

For antifungal formulations, device selection is particularly relevant because nebulization may affect not only aerosol output but also formulation integrity. This consideration is especially important for lipid-based and liposomal preparations, for which excessive mechanical stress, heating, or prolonged recirculation may potentially alter pharmaceutical characteristics. Consequently, aerosol delivery should be considered an integrated formulation–device process rather than a simple method of administration.

### 5.1 Nebulizer technologies

Three major nebulizer technologies are used for pulmonary drug delivery: jet nebulizers, ultrasonic nebulizers, and vibrating mesh nebulizers (VMNs).

Jet nebulizers generate aerosol using compressed gas and are widely available, but their performance may be affected by residual volume, variable aerosol output, prolonged treatment duration, and environmental aerosol release. These characteristics may contribute to variability in the effective pulmonary dose.

Ultrasonic nebulizers generate aerosol through high-frequency vibrations. Although they can provide relatively high aerosol output, heat generation during operation may be undesirable for formulations whose structural integrity is temperature-sensitive.

VMNs generate aerosol by passing the liquid formulation through a microperforated vibrating membrane. They generally provide low residual volume, efficient aerosol generation, limited heating, and the possibility of integration into respiratory circuits without introducing additional gas flow (24, 25, 34–37). These characteristics make VMNs technically attractive for aerosol administration of complex formulations, including liposomal preparations.

However, these pharmacotechnical advantages should not be interpreted as evidence of superior clinical efficacy. Comparative studies demonstrating improved patient-centered outcomes with one nebulizer technology over another remain limited. Device selection should therefore consider formulation compatibility, reproducibility of aerosol output, respiratory support configuration, institutional experience, and infection-control requirements.

### 5.2 Particle size and pulmonary deposition

Particle size is a major determinant of regional pulmonary deposition. Aerosol particles are commonly characterized by the mass median aerodynamic diameter (MMAD), which reflects their aerodynamic behavior within the respiratory tract.

Particles with an MMAD broadly within the respirable range of approximately 1–5 μm are generally considered suitable for lower-airway deposition. Larger particles are more likely to deposit in the upper airways through inertial impaction, whereas very small particles may remain suspended and be exhaled before deposition occurs.

Nevertheless, MMAD alone does not determine pulmonary drug delivery. Airway geometry, inspiratory flow, respiratory pattern, airway obstruction, secretion burden, regional ventilation, and underlying structural lung disease may substantially modify deposition. Consequently, an aerosol formulation with favorable *in vitro* particle characteristics may still show highly heterogeneous intrapulmonary distribution in patients with severe pulmonary disease.

This limitation is particularly relevant to fungal infections associated with cavitation, airway obstruction, consolidation, necrosis, or markedly heterogeneous ventilation. Effective aerosol generation therefore does not guarantee effective drug exposure within the pathological lesion.

### 5.3 Aerosol delivery during invasive mechanical ventilation

Aerosol administration during invasive mechanical ventilation presents additional technical challenges because the ventilator circuit, artificial airway, humidification system, and ventilatory parameters may substantially influence pulmonary deposition (24, 25, 34–37).

Drug loss may occur within the ventilator tubing, endotracheal tube, humidification system, and expiratory filter. Nebulizer position within the circuit, inspiratory flow pattern, tidal volume, respiratory rate, inspiratory time, and synchronization between aerosol generation and inspiratory flow may further influence the delivered dose.

VMNs offer practical advantages in mechanically ventilated patients because they can be incorporated into the ventilator circuit without introducing additional gas flow or substantially altering ventilator settings. Nevertheless, optimal device placement and circuit configuration remain important determinants of aerosol delivery.

Humidification represents an additional challenge because it may increase aerosol particle size and circuit deposition. However, interruption of active humidification solely to improve aerosol delivery should not be considered a routine strategy without consideration of airway safety, secretion management, and the patient's respiratory requirements.

Aerosolized antifungal administration during mechanical ventilation may also increase drug deposition on expiratory filters, potentially increasing airflow resistance. Filters should therefore be monitored and replaced according to device characteristics, institutional protocols, and manufacturer recommendations.

Importantly, most available evidence concerning aerosol optimization during mechanical ventilation derives from *in vitro*, bench, scintigraphic, or pharmacokinetic investigations rather than trials demonstrating improved clinical outcomes. Technical optimization should therefore be distinguished from demonstrated therapeutic efficacy.

### 5.4 Aerosol delivery during high-flow respiratory support

High-flow nasal oxygen (HFNO) has expanded opportunities for aerosol administration in spontaneously breathing patients requiring respiratory support (26–30). Aerosol can be introduced into the high-flow circuit, allowing drug delivery without interrupting oxygen therapy.

However, pulmonary deposition during HFNO is influenced by several interacting variables, including nebulizer type and position, gas flow, cannula size, nasal anatomy, mouth position, respiratory pattern, and the relationship between device flow and patient inspiratory flow (26–30).

Experimental and scintigraphic studies generally indicate that increasing high-flow rates may reduce the proportion of aerosol reaching the lower respiratory tract because of greater inertial impaction and upper-airway losses. Nevertheless, reduction of HFNO flow solely to increase aerosol deposition should not compromise oxygenation, respiratory support, or patient comfort. Clinical respiratory requirements must remain the primary determinant of HFNO settings.

Accordingly, although aerosol delivery through HFNO is technically feasible, the optimal strategy for antifungal administration remains incompletely defined, and evidence demonstrating improved antifungal outcomes is lacking.

### 5.5 Safety, device contamination, and reproducibility

Aerosol administration introduces safety and implementation considerations beyond drug pharmacology. Environmental dispersion may expose healthcare workers to aerosolized medication, particularly when open nebulization systems or poorly controlled expiratory pathways are used. Appropriate ventilation, personal protective measures when indicated, and institutional aerosol-safety procedures should therefore be considered.

Nebulizers and associated circuit components may also become contaminated if cleaning, handling, and replacement procedures are inadequate. Strict adherence to manufacturer instructions and institutional infection-control protocols is necessary, particularly in immunocompromised populations.

Another important issue is **formulation device compatibility**. Different nebulizers may produce substantially different aerosol outputs from the same nominal drug dose, and complex formulations may behave differently according to device characteristics. Consequently, nominal dose should not be assumed to correspond directly to pulmonary delivered dose.

Future clinical studies should therefore report the complete aerosol-delivery methodology, including formulation, concentration, nebulizer model, device position, respiratory support configuration, humidification conditions, treatment duration, and, whenever possible, measurements or estimates of delivered pulmonary dose. Standardization of these variables is essential for reproducibility and meaningful comparison across studies.

### 5.6 From technical feasibility to clinical effectiveness

Modern aerosol technologies have substantially improved the technical feasibility of pulmonary antifungal administration. However, successful aerosol generation and measurable pulmonary deposition represent only the first steps in establishing clinical effectiveness.

For inhaled antifungal therapy to provide meaningful benefit, the drug must be deposited within the anatomically relevant region, remain pharmacologically active, achieve adequate local exposure, and reach fungal organisms within the affected pulmonary compartment. These requirements may differ substantially according to the underlying fungal disease.

Therefore, evidence derived from aerosol engineering or pharmacokinetic studies should not be considered equivalent to evidence of therapeutic efficacy. Clinical benefit ultimately requires demonstration in appropriately designed studies evaluating disease-specific and patient-centered outcomes.

## 6 Clinical applications

The clinical relevance of aerosolized antifungal therapy varies substantially according to the underlying fungal disease, host characteristics, anatomical distribution of infection, and accessibility of the affected pulmonary compartment. Evidence supporting prophylactic use should therefore be clearly distinguished from evidence for treatment of established fungal disease. Similarly, pharmacological rationale and technical feasibility should not be interpreted as evidence of clinical efficacy.

At present, the strongest evidence supports aerosolized amphotericin B formulations for prevention of invasive pulmonary aspergillosis (IPA) in selected high-risk populations. Therapeutic applications are considerably less established and should be evaluated within a disease-specific framework.

### 6.1 Prophylaxis of invasive pulmonary aspergillosis during prolonged neutropenia

Prevention of IPA during prolonged neutropenia represents the clinical application supported by the strongest randomized evidence for aerosolized antifungal therapy. Patients with hematological malignancies and prolonged neutropenia are particularly susceptible to IPA because inhaled *Aspergillus* conidia establish infection initially within the respiratory tract.

Direct pulmonary administration therefore provides a biologically rational prophylactic approach by delivering antifungal drug to the primary site of fungal exposure while limiting systemic exposure.

The randomized, double-blind, placebo-controlled trial by Rijnders et al. evaluated aerosolized liposomal amphotericin B in 271 patients with prolonged neutropenia and demonstrated a significant reduction in proven or probable IPA compared with placebo (1). Subsequent reviews have supported the feasibility and generally favorable tolerability of this strategy while highlighting heterogeneity in dosing schedules, patient populations, aerosol-delivery techniques, and concomitant systemic prophylaxis (2–4).

Accordingly, prophylaxis against IPA in selected patients with prolonged neutropenia remains the best-supported clinical application of aerosolized antifungal therapy. Nevertheless, implementation varies among institutions, and aerosolized prophylaxis should be integrated into broader antifungal prevention strategies rather than considered universally applicable to all immunocompromised patients. Selected clinical evidence supporting aerosolized antifungal therapy and the corresponding hierarchy of evidence are summarized in Table 2.

**Table 2:** Key clinical evidence informing the development and use of aerosolized antifungal therapy.

Study	Design/sample size	Clinical setting	Intervention/exposure	Main findings	Nature of evidence	Interpretation
Rijnders et al. (2008) (1)	Randomized, double-blind, placebo-controlled trial; *n* = 271	Patients with hematologic disease and prolonged neutropenia	Aerosolized liposomal amphotericin B vs. placebo	Aerosolized L-AmB significantly reduced the incidence of proven or probable invasive pulmonary aspergillosis and was generally well tolerated	Randomized controlled evidence	Provides the strongest direct clinical evidence supporting aerosolized antifungal prophylaxis in selected high-risk patients
Agarwal et al. (2026) (43)	Multicenter, randomized, double-blind, parallel-group, placebo-controlled phase 2 trial; *n* = 43	Adults with asthma and allergic bronchopulmonary aspergillosis (ABPA)	Inhaled itraconazole (PUR1900) 20 mg (*n* = 18) or 40 mg (*n* = 16) once daily vs. placebo (*n* = 9) for 16 weeks	PUR1900 40 mg was associated with placebo-adjusted improvements in FEV_1_ and ACQ-7 and a reduction in serum total IgE; no comparable placebo-adjusted effects were observed with 20 mg. Adverse-event rates were similar across groups and no serious adverse events were reported	Randomized phase 2 clinical evidence; exploratory efficacy assessment	Provides proof-of-concept evidence for a dedicated inhaled triazole in ABPA and supports further clinical evaluation; the small sample size and absence of a prespecified primary efficacy endpoint preclude definitive efficacy conclusions
Hagiya et al. (2023) (3)	Systematic scoping review; 27 publications included	Multiple populations and clinical settings	Predominantly nebulized liposomal amphotericin B	Overall respiratory tolerability was generally favorable; accumulated evidence primarily supported safety, tolerability, and prophylactic use, particularly after lung transplantation, with substantial heterogeneity among studies	Evidence synthesis	Supports feasibility and short-term tolerability while highlighting heterogeneity in indications, dosing, delivery methods, and study quality
Vuong et al. (2023) (4)	Narrative review	Pulmonary fungal diseases and high-risk populations	Multiple inhaled antifungal formulations and delivery strategies	Summarized clinical applications, pharmacological rationale, technical challenges, and major evidence gaps	Narrative evidence synthesis	Supports the biological and pharmacological rationale for pulmonary delivery but does not establish therapeutic efficacy for investigational indications
Murray et al. (2020) (32)	Review of preclinical and early clinical development	Respiratory *Aspergillus* disease	Opelconazole (PC945), a triazole specifically developed for inhaled administration	Reported prolonged pulmonary exposure, limited systemic exposure, and preclinical/early clinical findings supporting continued development	Early translational and clinical-development evidence	Supports further investigation of a dedicated inhaled triazole but does not establish routine clinical use
Singh et al. (2023) (33)	Clinical report/limited clinical experience	Pseudomembranous tracheobronchial aspergillosis	Inhaled PC945/opelconazole as part of antifungal management	Reported clinical use of a lung-targeted triazole in airway-predominant aspergillosis	Limited clinical evidence	Provides hypothesis-generating support for targeted pulmonary therapy in anatomically airway-accessible disease; efficacy requires controlled evaluation

Summary of selected clinical evidence informing the development and use of aerosolized antifungal therapy. Randomized clinical evidence supports prophylaxis against invasive pulmonary aspergillosis in selected high-risk populations and, more recently, provides phase 2 proof-of-concept evidence for inhaled itraconazole (PUR1900) in allergic bronchopulmonary aspergillosis. Evidence for therapeutic applications in established invasive pulmonary fungal disease remains limited and derives predominantly from early clinical development, observational studies, and individual clinical reports. Pharmacological rationale, technical feasibility, and favorable pulmonary drug exposure should not be interpreted as evidence of therapeutic efficacy. ABPA, allergic bronchopulmonary aspergillosis; ACQ-7, Asthma Control Questionnaire-7; FEV_1_, forced expiratory volume in 1 s; IPA, invasive pulmonary aspergillosis; L-AmB, liposomal amphotericin B.

### 6.2 Prophylaxis after lung transplantation

Lung transplant recipients represent another population in which aerosolized amphotericin B formulations are frequently incorporated into antifungal prophylactic protocols. The transplanted lung is continuously exposed to inhaled fungal conidia, while impaired mucociliary clearance, airway ischemia, anastomotic abnormalities, immunosuppressive therapy, and repeated airway instrumentation increase susceptibility to *Aspergillus* infection.

Direct pulmonary delivery is therefore pharmacologically attractive, particularly during the early post-transplant period. Aerosolized amphotericin B deoxycholate, lipid complex, and liposomal formulations have been used in different institutional protocols, either alone or in combination with systemic antifungal agents (2–4).

However, unlike prophylaxis during prolonged neutropenia, evidence after lung transplantation is predominantly observational. Considerable heterogeneity exists regarding formulation, dose, frequency, duration, nebulization device, concomitant systemic prophylaxis, and criteria for discontinuation.

Recent IDSA guidance for adult solid organ transplant recipients further emphasizes that, when anti-*Aspergillus* prophylaxis or pre-emptive therapy is considered after lung transplantation, antifungal selection should be individualized according to tolerability, drug–drug interactions, feasibility of administration, cost, local resources, and epidemiological factors; no single prophylactic strategy is currently supported as universally optimal (42).

Aerosolized amphotericin B should therefore be regarded as a center specific prophylactic strategy supported mainly by observational evidence, rather than as a universally standardized approach.

### 6.3 Treatment of established invasive pulmonary aspergillosis and airway aspergillosis

The evidence supporting aerosolized antifungal therapy for treatment of established IPA is substantially weaker than that supporting prophylaxis. Systemic mold active antifungal therapy remains the standard of care because invasive aspergillosis may involve pulmonary parenchyma, blood vessels, and extrapulmonary sites (8).

Angioinvasion is particularly important when considering aerosolized therapy. Although inhaled drug may achieve high concentrations at airway and alveolar surfaces, aerosol deposition cannot ensure adequate exposure within vascularly invasive lesions, necrotic tissue, or extrapulmonary sites. Aerosolized therapy should therefore not replace systemic treatment for IPA.

A more plausible investigational role may exist in selected airway predominant forms of aspergillosis, including tracheobronchial disease, because fungal lesions are anatomically closer to the respiratory surface and potentially accessible to inhaled drug. This anatomical rationale has stimulated interest in inhaled amphotericin B and dedicated inhaled triazoles such as opelconazole (31–33).

However, available therapeutic evidence remains limited, and current data are insufficient to establish aerosolized antifungal therapy as standard treatment for airway aspergillosis. Its potential role should therefore be regarded as adjunctive and investigational, particularly when systemic treatment is limited by toxicity, drug interactions, or inadequate local response.

### 6.4 Allergic bronchopulmonary aspergillosis

Allergic bronchopulmonary aspergillosis (ABPA) represents a distinct airway-predominant, immune-mediated disease associated with hypersensitivity to Aspergillus antigens, most commonly occurring in patients with asthma or cystic fibrosis. Unlike invasive pulmonary aspergillosis, tissue invasion is not the defining pathological feature; instead, fungal persistence within the airways contributes to sustained antigenic exposure, inflammation, mucus impaction, and progressive structural lung damage. This airway-centered pathophysiology provides a particularly plausible rationale for targeted pulmonary antifungal delivery.

Recent randomized clinical evidence has strengthened this rationale. In the multicenter, randomized, double-blind, placebo-controlled phase 2 PUR1900-ABPA trial, 43 adults with asthma and ABPA received inhaled itraconazole (PUR1900) 20 mg, PUR1900 40 mg, or placebo once daily for 16 weeks. At week 16, the 40-mg group showed placebo-adjusted improvements in FEV_1_ and asthma control, together with a reduction in serum total IgE, while adverse-event rates were comparable across treatment groups (43).

These findings provide clinically relevant proof-of-concept evidence for targeted inhaled antifungal therapy in ABPA. However, the trial was exploratory, included a relatively small number of participants, and had no prespecified primary efficacy endpoint. The results therefore do not establish inhaled itraconazole as standard therapy for ABPA but support further adequately powered clinical evaluation. More broadly, ABPA illustrates a disease phenotype in which the anatomical localization of fungal burden within the airways may make targeted pulmonary delivery particularly attractive, while clinical benefit must still be demonstrated against established treatment strategies.

### 6.5 Chronic pulmonary aspergillosis

Chronic pulmonary aspergillosis (CPA) differs fundamentally from IPA in host characteristics, disease evolution, and pulmonary anatomy. CPA typically develops in patients with underlying structural lung disease rather than profound immunosuppression and is characterized by chronic pulmonary cavities, pleural thickening, progressive parenchymal destruction, fibrosis, and, in some patients, intracavitary fungal material (39, 40).

These characteristics create both a rationale and an important limitation for local antifungal delivery. Prolonged systemic antifungal therapy may be constrained by toxicity, drug–drug interactions, variable tolerability, and the need for extended treatment. Increasing local pulmonary exposure while limiting systemic exposure is therefore pharmacologically attractive.

However, the anatomical architecture of CPA may substantially limit aerosol effectiveness. Thick-walled cavities, mucus obstruction, distorted airways, fibrosis, heterogeneous ventilation, and intracavitary fungal masses may reduce or prevent deposition of inhaled particles within the pathological site. A cavity that communicates poorly with the bronchial tree may receive little aerosolized drug despite efficient nebulizer performance.

Consequently, the presence of a localized pulmonary lesion should not automatically be interpreted as evidence that the lesion is accessible to inhaled therapy. At present, clinical evidence supporting aerosolized antifungal therapy for CPA remains very limited, and systemic antifungal treatment continues to represent the principal pharmacological strategy.

CPA nevertheless remains an important area for future investigation because disease phenotypes differ considerably in airway communication, cavity architecture, fungal burden, and regional ventilation. Studies combining pulmonary imaging, aerosol-deposition assessment, and local pharmacokinetics may help determine whether selected anatomical phenotypes could eventually benefit from adjunctive inhaled therapy.

### 6.6 Pulmonary mucormycosis

Pulmonary mucormycosis is an aggressive invasive fungal infection occurring predominantly in patients with severe immunosuppression, hematological malignancy, uncontrolled diabetes, transplantation, or other major predisposing conditions. Its pathogenesis is characterized by marked angioinvasion, vascular thrombosis, tissue ischemia, and necrosis.

These features substantially limit the rationale for aerosolized therapy as a primary treatment. Although inhaled amphotericin B could theoretically increase drug exposure at accessible respiratory surfaces, aerosol delivery cannot reliably reach thrombosed vessels, deeply necrotic tissue, or extrapulmonary sites.

Systemic antifungal therapy and, when feasible, surgical management therefore remain central to treatment. Aerosolized amphotericin B should not be considered a substitute for systemic therapy in pulmonary mucormycosis.

Any potential adjunctive role would require prospective investigation and would need to demonstrate that local drug delivery adds clinically meaningful benefit to established systemic treatment. At present, evidence is insufficient to recommend routine aerosolized therapy for pulmonary mucormycosis.

### 6.7 Pulmonary cryptococcosis

Pulmonary cryptococcosis encompasses a broad clinical spectrum ranging from localized pulmonary nodules in immunocompetent individuals to severe pulmonary disease associated with disseminated or central nervous system infection in immunocompromised patients.

This biological heterogeneity is important when evaluating inhaled therapy. Localized pulmonary disease might appear theoretically suitable for targeted drug delivery; however, radiologically localized lesions are not necessarily accessible to inhaled particles. Nodular or parenchymal lesions may have limited airway communication, and pulmonary cryptococcosis may coexist with clinically occult extrapulmonary dissemination.

Because treatment decisions depend on host immune status, disease severity, and assessment for dissemination, systemic antifungal therapy remains the established approach when treatment is indicated. Current evidence does not support routine aerosolized antifungal therapy for pulmonary cryptococcosis.

Accordingly, the rationale for inhaled therapy in cryptococcosis remains weak unless future studies identify specific airway-accessible phenotypes in which pulmonary drug delivery provides a measurable advantage without compromising systemic disease management.

### 6.8 Pneumocystis jirovecii pneumonia

*Pneumocystis jirovecii* pneumonia (PJP) represents a distinct example because inhaled antimicrobial delivery has an established historical role in prophylaxis, particularly with aerosolized pentamidine. However, this experience should not be extrapolated directly to aerosolized antifungal therapy.

PJP is typically characterized by diffuse bilateral pulmonary involvement rather than localized airway-accessible fungal lesions. Effective treatment therefore requires adequate drug exposure throughout extensively affected alveolar regions, and systemic trimethoprim–sulfamethoxazole remains the standard therapeutic approach.

Although the diffuse alveolar distribution of PJP might theoretically permit aerosol deposition, severe respiratory disease is frequently associated with heterogeneous ventilation, hypoxemia, and impaired distal deposition. Furthermore, evidence supporting aerosolized conventional antifungal agents for treatment of PJP is lacking.

PJP therefore illustrates an important principle: technical accessibility of the respiratory tract does not itself establish a therapeutic role for aerosolized antifungal drugs. Pathogen susceptibility, disease biology, and evidence of clinical efficacy remain decisive.

### 6.9 Endemic pulmonary mycoses

Endemic fungal infections, including histoplasmosis, blastomycosis, and coccidioidomycosis, may produce pulmonary manifestations ranging from self-limited infection to chronic cavitary disease, diffuse pneumonia, and extrapulmonary dissemination.

Despite pulmonary involvement, these diseases generally do not provide a strong current rationale for aerosolized antifungal therapy. Infection may extend beyond airway-accessible surfaces, and systemic dissemination may occur depending on the pathogen and host immune status. In chronic cavitary disease, the same anatomical limitations affecting aerosol deposition in CPA—including poor cavity communication, fibrosis, structural distortion, and heterogeneous ventilation—may also apply.

Systemic antifungal therapy therefore remains the standard pharmacological approach when treatment is indicated. At present, clinical evidence is insufficient to support routine inhaled antifungal therapy for endemic pulmonary mycoses.

Future research would need to define disease-specific pulmonary phenotypes, demonstrate effective local deposition at the pathological site, and establish a clinical advantage over systemic therapy before inhaled approaches could be considered.

### 6.10 Disease-Specific interpretation of aerosolized antifungal therapy

The available evidence demonstrates that the potential role of aerosolized antifungal therapy cannot be generalized across pulmonary fungal diseases and Aspergillus-associated conditions. Its clinical relevance depends on the interaction among pathogen biology, disease phenotype, anatomical distribution, regional ventilation, accessibility of the affected pulmonary compartment, host characteristics, and the strength of available clinical evidence.

The strongest established evidence supports prophylaxis of invasive pulmonary aspergillosis in selected high-risk patients, particularly during prolonged neutropenia, whereas prophylactic use after lung transplantation remains center-dependent and supported predominantly by observational evidence. More recently, randomized phase 2 evidence with inhaled itraconazole (PUR1900) has provided proof-of-concept support for targeted pulmonary antifungal delivery in allergic bronchopulmonary aspergillosis (ABPA), an airway-predominant, immune-mediated Aspergillus-associated disease (43). However, these findings remain exploratory and do not establish inhaled itraconazole as standard therapy for ABPA.

For established invasive pulmonary aspergillosis, airway/tracheobronchial aspergillosis, and chronic pulmonary aspergillosis, inhaled antifungal therapy remains investigational and, where considered, should generally be regarded as an adjunct rather than a replacement for established systemic antifungal treatment. The potential for benefit is likely to be greatest in anatomically accessible airway-predominant disease and lowest in settings characterized by angioinvasion, necrosis, poor ventilation, limited airway communication, or extrapulmonary dissemination.

For pulmonary mucormycosis, cryptococcosis, Pneumocystis jirovecii pneumonia, and endemic pulmonary mycoses, current evidence does not support routine aerosolized antifungal therapy. In these conditions, disease-specific factors including angioinvasion, diffuse alveolar involvement, parenchymal or nodular localization, potential dissemination, and limited accessibility of pathological lesions may substantially reduce the potential advantage of local pulmonary drug delivery.

Accordingly, future investigation should move away from a uniform “pulmonary fungal disease” model and instead adopt a disease- and phenotype-specific approach. Clinical development should integrate pathogen susceptibility, anatomical accessibility, regional ventilation, pulmonary pharmacokinetics and pharmacodynamics, host characteristics, formulation–device performance, and clinically meaningful outcomes. The disease-specific rationale, current level of evidence, major anatomical and biological limitations, and principal research priorities are summarized in Table 3.

**Table 3:** Disease-oriented framework for the current and potential role of aerosolized antifungal therapy.

Clinical condition	Current role of aerosolized antifungal therapy	Rationale for pulmonary delivery	Major disease-specific limitations	Current evidence level/interpretation	Key research needs
IPA prophylaxis during prolonged neutropenia	Evidence-supported prophylactic application in selected high-risk patients	Direct delivery to the respiratory tract, the initial site of *Aspergillus* exposure; high local drug concentrations with limited systemic exposure	Heterogeneity in dosing schedules, nebulization protocols, patient selection, and concomitant systemic prophylaxis	Strongest established clinical evidence, including randomized placebo-controlled data for aerosolized L-AmB (1–4)	Standardized dosing and delivery protocols; comparative effectiveness studies; identification of patients deriving greatest benefit
Lung transplantation—*Aspergillus* prophylaxis	Center-specific prophylactic strategy; not universally standardized	Direct exposure of the transplanted lung; impaired mucociliary clearance and airway/anastomotic susceptibility provide a biological rationale for local prophylaxis	Heterogeneous formulations, dosing schedules, duration, devices, and concomitant systemic prophylaxis	Predominantly observational evidence; no single prophylactic strategy is universally supported (2–4, 42)	Comparative prophylactic strategies; optimal formulation, dose, duration, and patient selection
Established invasive pulmonary aspergillosis (IPA)	Investigational adjunct; not a replacement for systemic therapy	May increase antifungal exposure at airway-accessible pulmonary surfaces	Angioinvasion, vascular involvement, necrosis, consolidation, heterogeneous ventilation, and potential extrapulmonary dissemination limit the effectiveness of local delivery	Insufficient therapeutic evidence; systemic mold-active therapy remains standard of care (8)	Randomized studies of adjunctive inhaled therapy; regional PK/PD and lesion-deposition studies; phenotype-based patient selection
Airway/tracheobronchial aspergillosis	Potential investigational adjunct	Airway-predominant lesions are anatomically more accessible to inhaled drug than deeply parenchymal or vascular lesions	Variable depth and extent of airway invasion; limited controlled clinical data	Limited clinical and early developmental evidence, including experience with inhaled triazoles (31–33)	Prospective controlled trials; bronchoscopic and microbiological endpoints; comparison with systemic therapy alone
Allergic bronchopulmonary aspergillosis (ABPA)	Investigational; emerging randomized phase 2 evidence	ABPA is an airway-predominant, immune-mediated *Aspergillus*-associated disease. Targeted inhaled delivery may increase local airway antifungal exposure while limiting systemic exposure and drug–drug interactions associated with oral azoles	ABPA is primarily an immune-mediated rather than invasive fungal disease; reducing airway fungal burden addresses only one component of its pathophysiology. Available randomized evidence remains exploratory and derives from a small phase 2 trial	Randomized phase 2 proof-of-concept evidence. PUR1900 40 mg showed encouraging effects on lung function and asthma control with a favorable safety profile, but the study was small and had no prespecified primary efficacy endpoint (43)	Larger adequately powered confirmatory trials; predefined clinically meaningful endpoints; exacerbation and corticosteroid-sparing outcomes; durability of response; long-term safety; identification of patients most likely to benefit
Chronic pulmonary aspergillosis (CPA)	Investigational; no established routine role	Chronic localized pulmonary disease and prolonged treatment requirements provide a theoretical rationale for targeted pulmonary delivery	Thick-walled cavities, intracavitary fungal material, fibrosis, airway distortion, mucus obstruction, heterogeneous ventilation, and variable cavity–airway communication may markedly limit aerosol deposition	Very limited evidence for inhaled therapy; systemic antifungal therapy remains the established pharmacological approach (39, 40)	Identification of aerosol-accessible CPA phenotypes; imaging/deposition studies; intracavitary and regional PK; controlled adjunctive trials
Pulmonary mucormycosis	No established role; routine aerosolized therapy unsupported	Theoretical possibility of increasing local amphotericin B exposure	Marked angioinvasion, vascular thrombosis, ischemia, tissue necrosis, and possible dissemination substantially limit local aerosol delivery	Insufficient evidence; systemic antifungal therapy ± surgery remains central	Carefully designed studies of adjunctive local therapy only where biologically justified; demonstration of drug penetration into affected lesions
Pulmonary cryptococcosis	No established role	Localized pulmonary involvement could theoretically permit targeted delivery in selected phenotypes	Nodular or parenchymal lesions may be poorly airway-accessible; potential occult extrapulmonary or CNS dissemination requires systemic assessment and treatment	Insufficient evidence for aerosolized therapy	Definition of potentially airway-accessible phenotypes; pulmonary deposition and PK studies before therapeutic trials
*Pneumocystis jirovecii* pneumonia (PJP)	No established role for aerosolized conventional antifungal therapy	Diffuse pulmonary distribution theoretically permits respiratory drug exposure; aerosolized pentamidine has historical relevance for prophylaxis	Diffuse alveolar disease, severe hypoxemia, heterogeneous ventilation, and absence of evidence supporting aerosolized conventional antifungal agents for treatment	No evidence supporting routine aerosolized conventional antifungal treatment	No current basis for conventional inhaled antifungal trials without compelling preclinical and pharmacological rationale
Endemic pulmonary mycoses	No established role	Selected chronic pulmonary phenotypes might theoretically appear amenable to local delivery	Disease may be parenchymal, cavitary, multifocal, or disseminated; structural distortion and poor cavity–airway communication may limit deposition	Insufficient clinical evidence; systemic therapy remains standard when treatment is indicated	Disease-specific assessment of anatomical accessibility and pulmonary PK before clinical evaluation

Disease-oriented interpretation of the current and potential roles of aerosolized antifungal therapy across major pulmonary fungal and *Aspergillus*-associated diseases. The potential clinical value of pulmonary antifungal delivery depends on disease pathophysiology, anatomical distribution, accessibility of the affected pulmonary compartment, regional ventilation, antifungal pharmacology, and the strength of available clinical evidence. The strongest established evidence supports prophylaxis of invasive pulmonary aspergillosis in selected high-risk populations. More recently, randomized phase 2 evidence with inhaled itraconazole (PUR1900) has provided proof-of-concept support for targeted pulmonary antifungal delivery in allergic bronchopulmonary aspergillosis, although this approach remains investigational. Therapeutic applications in established invasive pulmonary fungal disease remain investigational or unsupported and should not replace guideline-recommended systemic antifungal therapy when invasive, angioinvasive, disseminated, or otherwise systemically relevant disease is present. ABPA, allergic bronchopulmonary aspergillosis; ACQ-7, Asthma Control Questionnaire-7; CNS, central nervous system; CPA, chronic pulmonary aspergillosis; FEV_1_, forced expiratory volume in 1 s; IPA, invasive pulmonary aspergillosis; L-AmB, liposomal amphotericin B; PJP, *Pneumocystis jirovecii* pneumonia; PK/PD, pharmacokinetics/pharmacodynamics.

## 7 Current limitations and future perspectives

Despite advances in antifungal formulations and aerosol-delivery technologies, several scientific, clinical, and technical limitations continue to restrict the broader implementation of aerosolized antifungal therapy. Most importantly, the ability to generate an efficient aerosol and achieve high respiratory drug concentrations does not necessarily translate into effective exposure at the site of fungal disease or into improved clinical outcomes. Future research should therefore move from generalized concepts of pulmonary drug delivery toward disease- and phenotype-specific evaluation.

### 7.1 Limited and uneven clinical evidence

The quality of evidence supporting aerosolized antifungal therapy varies substantially according to clinical indication. The strongest evidence concerns prophylaxis of invasive pulmonary aspergillosis (IPA) in selected high-risk populations, particularly patients with prolonged neutropenia (1–4). Aerosolized amphotericin B formulations are also used after lung transplantation, although protocols remain heterogeneous and the supporting evidence is predominantly observational.

By contrast, evidence for treatment of established pulmonary fungal disease remains limited. Available therapeutic data derive mainly from pharmacokinetic studies, small observational series, early-phase investigations, and case reports. These data may establish biological plausibility, technical feasibility, or preliminary safety, but they cannot demonstrate therapeutic efficacy.

Large, adequately powered randomized controlled trials evaluating clinically meaningful outcomes are therefore required before therapeutic indications for aerosolized antifungal agents can be expanded beyond currently supported settings.

### 7.2 Disease phenotype and anatomical accessibility

A major challenge is identifying pulmonary fungal disease phenotypes in which inhaled drug can actually reach the pathological site.

The term “pulmonary fungal disease” encompasses conditions with markedly different anatomical distributions. Airway-predominant disease may be relatively accessible to aerosolized drug, whereas angioinvasive lesions, extensive consolidation, necrotic tissue, thick-walled cavities, mucus-obstructed regions, pleural disease, and extrapulmonary dissemination may be poorly accessible or inaccessible through the respiratory route.

Even within the same disease, anatomical accessibility may vary considerably. In chronic pulmonary aspergillosis, for example, communication between cavities and the bronchial tree, regional ventilation, intracavitary material, fibrosis, and airway distortion may determine whether meaningful aerosol deposition is achievable.

Future studies should therefore integrate high-resolution imaging, characterization of lesion–airway communication, regional ventilation assessment, and, where feasible, quantitative measurements of pulmonary drug deposition. Anatomical phenotype may ultimately prove as important as fungal species in determining whether inhaled therapy is pharmacologically rational.

### 7.3 Patient selection and biomarkers

No validated biomarkers currently identify patients with established pulmonary fungal disease who are likely to benefit specifically from adjunctive aerosolized antifungal therapy.

Future patient-selection strategies should combine conventional microbiology with quantitative fungal burden, fungal biomarkers, molecular diagnostics, pulmonary imaging, host immune profiling, and respiratory physiology. Pharmacokinetic and pharmacodynamic measurements should also be incorporated whenever possible to determine whether adequate drug exposure is achieved within the relevant pulmonary compartment.

Such approaches may help distinguish patients in whom local pulmonary drug delivery offers a genuine pharmacological advantage from those in whom systemic treatment alone remains more appropriate.

### 7.4 Standardization of aerosol delivery and pulmonary dose

The absence of standardized aerosol-delivery protocols remains a major limitation. Pulmonary deposition is influenced by formulation concentration, nebulizer technology, particle-size distribution, device position, respiratory pattern, airway obstruction, humidification, ventilator settings, HFNO flow, and underlying lung disease (24–30, 34–37).

Consequently, identical nominal doses may result in substantially different pulmonary delivered doses across devices and clinical conditions. This variability complicates comparison among published studies and may contribute to inconsistent clinical results.

Future trials should report aerosol methodology in sufficient detail to permit reproducibility, including formulation, concentration, nebulizer model, device configuration, respiratory support settings, humidification conditions, administration time, and, whenever feasible, estimated or measured pulmonary delivered dose.

Development of standardized formulation–device combinations may ultimately be necessary for reliable clinical implementation.

### 7.5 Safety, occupational exposure, and infection control

Although aerosolized liposomal amphotericin B has generally demonstrated favorable short-term respiratory tolerability, the safety of prolonged or repeated administration remains incompletely characterized.

Potential local adverse effects include cough, bronchospasm, airway irritation, and changes in respiratory mechanics. The long-term effects of repeated antifungal exposure on airway epithelium, local immune homeostasis, pulmonary microbiota and mycobiota, and selection of antifungal resistance remain uncertain.

Aerosol administration also raises practical safety considerations. Environmental dispersion may result in occupational exposure of healthcare personnel, while inadequate device handling may contribute to nebulizer or circuit contamination. Appropriate infection-control procedures, environmental precautions, device cleaning or replacement protocols, and adherence to manufacturer recommendations are therefore essential.

Particular caution is required when intravenous formulations are repurposed for inhalation because formulation–device compatibility, aerosol characteristics, and reproducibility of the delivered dose may not have undergone the regulatory evaluation required for a dedicated inhaled medicinal product.

### 7.6 Emerging inhaled antifungal agents

The development of antifungal formulations specifically designed for pulmonary administration represents an important area of translational research. Dedicated inhaled formulations may improve pulmonary drug exposure, reduce systemic exposure, and optimize formulation–device performance compared with aerosolization of preparations originally developed for systemic administration. However, these pharmacological and technological advantages should not be interpreted as evidence of clinical efficacy.

Recent clinical development of inhaled triazoles illustrates this evolving field. PUR1900, a dry-powder formulation of itraconazole specifically developed for pulmonary delivery, has recently provided randomized phase 2 proof-of-concept evidence in adults with asthma and allergic bronchopulmonary aspergillosis (ABPA) (43). In the PUR1900-ABPA trial, the 40-mg dose showed encouraging effects on lung function and asthma control together with a favorable safety profile. Nevertheless, the relatively small sample size, exploratory design, and absence of a prespecified primary efficacy endpoint require confirmation in larger, adequately powered trials before inhaled itraconazole can be considered an established therapeutic option for ABPA.

Opelconazole (PC945) represents another advanced inhaled triazole specifically designed for pulmonary administration. Preclinical and early clinical studies have demonstrated prolonged pulmonary exposure with limited systemic absorption, supporting continued investigation in respiratory Aspergillus disease (31–33). However, controlled clinical evidence remains limited, and its therapeutic role has not yet been established.

Other approaches include inhaled posaconazole formulations, such as nanocrystal-based dry-powder systems, which have demonstrated favorable pulmonary pharmacokinetic characteristics in preclinical models (41). These findings support further pharmaceutical and translational development but do not currently establish a clinical indication. Development of inhaled echinocandins and other novel antifungal formulations remains at an earlier stage, with insufficient clinical evidence to define a therapeutic role.

Overall, emerging inhaled antifungal agents should be evaluated according to a development pathway that integrates formulation stability, aerosol performance, reproducibility of delivered dose, regional pulmonary deposition, pharmacokinetics and pharmacodynamics, safety, and ultimately disease-specific clinical efficacy. Randomized phase 2 evidence with PUR1900 represents an important advance in this field, but adequately powered confirmatory trials with predefined clinically meaningful endpoints remain necessary before dedicated inhaled antifungal formulations can be incorporated into routine therapeutic practice.

### 7.7 Future research priorities

Future investigation should move beyond demonstrating aerosol feasibility or microbiological clearance alone and determine whether targeted pulmonary antifungal delivery improves outcomes that are meaningful to patients.

Several priorities emerge from the current evidence:

- disease and phenotype-specific randomized trials of adjunctive aerosolized therapy;
- pulmonary pharmacokinetic/pharmacodynamic studies linking administered dose to regional drug exposure;
- imaging and deposition studies evaluating whether inhaled drug reaches fungal lesions;
- development of biomarkers and quantitative microbiological approaches for patient selection;
- standardized formulation device combinations and reproducible dosing protocols;
- evaluation of dedicated inhaled antifungal compounds;
- assessment of long-term respiratory safety, microbiome and mycobiome effects, occupational exposure, and antifungal resistance;
- incorporation of patient centered outcomes, including respiratory recovery, duration of hospitalization, quality of life, treatment tolerability, and health economic endpoints.

An additional, but clearly hypothesis-generating, area concerns the biological significance of persistent fungal airway carriage in selected critically ill patients. Experimental studies indicate that opportunistic yeasts such as *Nakaseomyces glabrata* can form biofilms, persist within macrophages, adapt to oxidative and metabolic stress, and evade host immune responses (15–23). These observations provide mechanistic models of fungal persistence but do not establish clinically relevant airway disease or an indication for antifungal treatment. Respiratory isolation of *Candida* species should continue to be interpreted according to established clinical and guideline criteria (5, 7, 9, 10). Prospective studies would be required to determine whether any reproducible phenotype of biologically active fungal airway persistence exists and, only subsequently, whether targeted pulmonary therapy could have clinical relevance.

More broadly, future trials should avoid treating pulmonary fungal diseases as a homogeneous therapeutic category. The probability of benefit from aerosolized therapy is likely to depend on the interaction among pathogen susceptibility, disease phenotype, lesion accessibility, regional ventilation, host characteristics, systemic antifungal exposure, and aerosol pharmacokinetics.

### 7.8 Translational perspective

The future role of aerosolized antifungal therapy will depend on demonstrating a clinically meaningful advantage over existing systemic strategies rather than simply achieving high pulmonary drug concentrations.

A precision approach to pulmonary antifungal delivery should therefore integrate the right drug, the right formulation, the right aerosol device, the right anatomical target, and the right patient. For some indications most notably prophylaxis of IPA in selected high-risk populations this concept already has clinical support. For treatment of established pulmonary fungal disease, however, it remains largely investigational.

The next generation of studies should therefore connect aerosol engineering and pulmonary pharmacology with disease-specific biology and clinically relevant outcomes. Only through this integrated approach can the potential role of aerosolized antifungal therapy within contemporary antifungal management be defined.

## 8 Conclusion

Aerosolized antifungal therapy provides a pharmacologically attractive strategy for increasing local pulmonary drug exposure while limiting systemic exposure. Among currently available formulations, liposomal amphotericin B has the most extensive clinical experience, with the strongest established evidence supporting prophylaxis of invasive pulmonary aspergillosis in selected high-risk populations, particularly patients with prolonged neutropenia. Aerosolized amphotericin B formulations are also incorporated into prophylactic strategies after lung transplantation, although protocols remain heterogeneous and the supporting evidence is predominantly observational.

Recent randomized phase 2 evidence with inhaled itraconazole (PUR1900) in allergic bronchopulmonary aspergillosis provides additional proof-of-concept support for disease-specific pulmonary antifungal delivery, particularly in an airway-predominant Aspergillus-associated condition (43). However, these findings remain exploratory and require confirmation in larger, adequately powered trials with predefined clinically meaningful endpoints before inhaled itraconazole can be incorporated into routine management of ABPA.

Beyond these settings, therapeutic applications of aerosolized antifungal therapy remain investigational. Their potential value cannot be generalized across pulmonary fungal diseases but depends on pathogen biology, disease phenotype, anatomical distribution, regional ventilation, and accessibility of the affected compartment. In established invasive pulmonary aspergillosis, airway/tracheobronchial aspergillosis, and chronic pulmonary aspergillosis, inhaled therapy may warrant further investigation as an adjunctive strategy but should not replace established systemic antifungal treatment. For pulmonary mucormycosis, cryptococcosis, Pneumocystis jirovecii pneumonia, and endemic pulmonary mycoses, current evidence is insufficient to support routine aerosolized antifungal therapy.

Advances in dedicated inhaled formulations and aerosol-delivery technologies may expand future therapeutic opportunities, but technical feasibility, high local drug concentrations, and favorable pulmonary pharmacokinetics should not be equated with clinical efficacy. Future studies should therefore adopt disease- and phenotype-specific approaches integrating pulmonary pharmacokinetics and pharmacodynamics, quantitative drug deposition, anatomical accessibility, formulation–device compatibility and reproducibility, long-term safety, and clinically meaningful patient outcomes.

Ultimately, the future role of aerosolized antifungal therapy will depend on identifying the diseases and patient phenotypes in which targeted pulmonary delivery provides a measurable clinical advantage over existing treatment strategies. Well-designed translational studies and adequately powered controlled trials are required before therapeutic indications can be expanded beyond currently supported or emerging proof-of-concept settings.
